# C-spine mutations of protein kinase C and Akt as a novel generalizable approach to create stable pseudokinases

**DOI:** 10.1016/j.jbc.2025.110921

**Published:** 2025-11-07

**Authors:** Stefanie J. Hodapp, Tiffany H. Kao, Jian Wu, Nileeka Balasuriya, Corina E. Antal, Susan S. Taylor, Alexandra C. Newton

**Affiliations:** 1 Department of Pharmacology, University of California, San Diego, La Jolla, California, USA; 2 Biomedical Sciences Graduate Program, University of California, San Diego, La Jolla, California, USA; 3 Moores Cancer Center, University of California San Diego, La Jolla, California, USA; 4 Department of Biochemistry and Molecular Biophysics, University of California San Diego, La Jolla, California, USA

**Keywords:** Akt, C-spine, kinase-dead, protein kinase C, pseudokinase

## Abstract

Protein kinases function not only through their catalytic phospho-transfer activity but also by noncatalytic scaffold mechanisms. Introduction of mutations to inactivate catalysis provides a tool to differentiate between the two; however, kinase-inactivating mutations may alter the structure of the kinase domain and perturb scaffold functions. Here, we developed a strategy that prevents ATP binding, thereby preventing catalysis, while stabilizing the active conformation of the kinase domain. This approach leverages the structural role of ATP in assembling the catalytic spine (C-spine), a hydrophobic core essential for the active conformation. Specifically, we substituted Val or Ala residues proximal to the binding position of the adenosine ring of ATP with Phe in three protein kinase C isozymes (PKCβII, γ, and θ) and Akt1. Structural modeling suggests that Phe substitutions at these positions are a surrogate for the adenosine ring of ATP to assemble the C-spine. Live-cell imaging using genetically encoded PKC and Akt activity reporters reveals that C-spine mutations abolish kinase activity. Furthermore, phosphorylation of the hydrophobic motif, an autophosphorylation site, is abolished in C-spine mutants of PKC family members and reduced in C-spine mutants of Akt1, independent of epidermal growth factor stimulation. In PKCβII, these C-spine mutations accelerate plasma membrane translocation, consistent with impaired autoinhibition due to the lack of hydrophobic motif phosphorylation. Despite adopting reduced autoinhibition, turnover experiments with PKCθ reveal C-spine mutants do not impair the stability of the full-length PKC. The generation of pseudokinases by C-spine mutations provides a generalizable strategy for elucidating noncatalytic kinase functions.

Dysregulation of kinases has been associated with a multitude of pathologies, including cancer and neurodegeneration (1, 2). One common approach to understand the role of a kinase in a disease context is to suppress the kinase activity through introduction of kinase-dead mutations (3, 4). Most kinase-dead mutations mutate one of the regulatory triad residues (Lys72, Glu91, and Asp184 in protein kinase A, PKA). The most common mutation, Lys72 to Ala, was first reported in Src and Fyn to confirm that this residue, identified by affinity labeling of the PKA C-subunit (5), was essential for catalytic activity (6, 7). Although the K72 mutant in Src can still bind ATP, it is kinase-dead (8). Lys72 can be substituted with other residues to create a kinase-dead phenotype (9), but mutations at this residue may compromise the structure of the kinase domain resulting in altered protein interactions as a secondary effect (10). Thus, kinase-dead mutations of the same kinase have yielded different results (11, 12). Identifying a common mutation that maintains the ATP-bound active structure of a kinase but prevents catalysis would be of great value in dissecting the role of catalysis *versus* scaffold functions.

The AGC branch of the kinome comprises approximately 60 evolutionarily related serine/threonine kinases that include the highly studied families, PKA, Akt (also known as protein kinase B (PKB)), and protein kinase C (PKC) (13). Members of these families each have unique regulatory mechanisms that dictate activation, either by regulatory domains in the same polypeptide that bind activators (*e.g.* PKC and Akt) or by having regulatory subunits that bind activators (*e.g.* PKA). The kinase domain is a highly conserved region across eukaryotic protein kinases and catalyzes the transfer of phosphate from ATP to protein substrates. Structurally, it consists of two lobes: the N-terminal lobe (N-lobe) and the C-terminal lobe (C-lobe) (14, 15, 16, 17). The smaller N-lobe, made up of β-sheets, plays a key role in nucleotide orientation and recognition, whereas the C-lobe, composed of α-helices, houses the substrate binding pocket (14, 15, 16, 17). These lobes are connected by a short loop, and the cleft between them serves as the site where ATP binding and phosphate transfer occur (14, 15, 16, 17). The kinase domain contains two key structural elements: the catalytic (C) spine and the regulatory (R) spine, collectively known as the hydrophobic spines (14, 15, 16, 17). The assembly of the R-spine, which is kinase-specific and typically driven by the phosphorylation of the activation loop, induces the active conformation of the enzyme by assembling the regulatory triad (K72, E91, and D184 in PKA) and is essential for optimal catalysis of heterologous substrates. In contrast, the C-spine is formed upon binding of the adenine ring of ATP, positioning the N- and C-lobes for catalysis (14, 15, 16, 17). Capping of the adenine ring of ATP in a hydrophobic shell is mediated almost entirely by N-lobe residues that include two highly conserved amino acids (V57 in the β2 strand and A70 in the β3 strand). Only one capping residue, L173, comes from the C-lobe, and it is always hydrophobic (18). Hu et al. previously discovered that mutation of the highly conserved C-spine alanine in the kinase domain of kinase suppressor of Ras (KSR1) to phenylalanine (A587F) blocked ATP binding but maintained the closed, active conformation of the kinase domain by completion of the C-spine (19). The discovery of this mutation allowed for the separation of the two functions of a kinase, catalytic and scaffolding, and unveiled a key role of KSR1 as a protein scaffold to activate Raf. To test the general applicability of this mutation, we made parallel mutations in two AGC kinase families: PKC and Akt.

The PKC family consists of nine isozymes that are divided into three different classes: conventional (α, β, and γ), novel (δ, θ, ε, and η), and atypical (ɩ and ζ) (20). The kinase activity of all members is allosterically regulated by N-terminal modules, including a pseudosubstrate sequence that blocks the active site in the absence of appropriate second messengers. Conventional and novel PKC isozymes also have two tandem C1 domains (C1A and C1B, of which the latter binds diacylglycerol in the mature enzyme) and a C2 domain (which in conventional PKC isozymes binds Ca^2+^), followed by the AGC kinase domain and a regulatory C-terminal tail. Newly synthesized PKC undergoes a series of priming phosphorylations (1) at the turn motif and, for mTORC2-dependent isozymes, the newly-identified TOR interaction motif (TIM), in the C-terminal tail catalyzed by mTORC2, (2) at the activation loop, near the entrance to the active site, catalyzed by PDK1, and (3) culminating in intramolecular autophosphorylation at a second site in the C-terminal tail referred to as the hydrophobic motif (21, 22, 23, 24, 25). This last phosphorylation of the hydrophobic motif is necessary for primed PKC to adopt an autoinhibited and phosphatase-resistant conformation (26). Disease-associated mutations in PKC that are unable to be phosphorylated at these three sites, or that are unable to adopt the autoinhibited conformation to protect these sites, are in a phosphatase-labile conformation that is rapidly degraded by the proteasome (26, 27, 28). In addition, without the priming phosphates, the membrane targeting modules are unmasked, resulting in enhanced membrane binding (29). Primed and autoinhibited PKC is activated following hydrolysis of phosphatidylinositol 4,5-bisphosphate (PIP_2_) which increases diacylglycerol (DG) and intracellular Ca^2+^ (30). These second messengers recruit PKC to the plasma membrane, where the C1B domain engages with membrane-bound DG resulting in a conformational change that releases the pseudosubstrate to enable downstream signaling (30).

The Akt family of kinases (Akt1, Akt2, and Akt3) are downstream effectors of phosphoinositide 3-kinase (PI3K) (31) by virtue of an N-terminal pleckstrin homology (PH) domain that binds phosphatidylinositol 3,4,5-trisphosphate (PIP_3_) and phosphatidylinositol 3,4-bisphosphate (32). Akt shares the same phosphorylations as PKC on the TIM and turn motif, the activation loop, and the hydrophobic motif, but they serve different functions (25, 31, 33, 34). Notably, whereas phosphorylation of the hydrophobic motif is constitutive and regulates the chronic stability of PKC, phosphorylation at the hydrophobic motif does not control the stability of Akt and, instead, controls acute activity (26). Except for phosphorylation at the TIM/turn motif, which occurs cotranslationally by mTORC2 (25, 35), phosphorylation at the activation loop by PDK1 and the hydrophobic motif occurs after agonist stimulation (25, 35, 36, 37). Specifically, activation of PI3K causes Akt to engage with PIP_3_ at the plasma membrane through its PH domain. This binding event releases the autoinhibitory constraints of the PH domain, exposing the activation loop for phosphorylation by PDK1, leading to activation of Akt (38). Although phosphorylation of the activation loop (Thr308 in Akt1) is necessary and sufficient for maximal activity in cells (34), phosphorylation at the hydrophobic motif (Ser473 in Akt1) fine tunes activity and substrate specificity (39). Numerous kinases have been proposed to phosphorylate the hydrophobic motif (40, 41, 42), most notably mTORC2 (43) and Akt itself (44). Whereas the hydrophobic motif is not phosphorylated in cells lacking functional mTORC2 (43), phosphorylation is effectively rescued by overexpression of PDK1 by a mechanism that depends on the intrinsic catalytic activity of Akt itself (25). This suggests that mTORC2, through phosphorylation of the TIM and turn motif, facilitates the necessary phosphorylation by PDK1, in turn promoting autophosphorylation (25, 45). In summary, whereas PKC is constitutively phosphorylated and has a pseudosubstrate for autoinhibition, Akt does not have a pseudosubstrate and depends on acute activation loop phosphorylation for activity. Kinase-dead constructs of both families of kinases have been extensively used to dissect their roles in health and disease, thus identifying mutations that maintain the ATP-bound active conformation, with an intact C-spine, would be of great value.

Here, we analyze two C-spine mutants (V356F and A369F in PKCβII) across several kinases, including the conventional PKCs, βII, and γ, the novel PKCθ, and Akt1. Molecular modeling reveals that mutation to phenylalanine mimics the adenine ring of ATP to align the C-spine. Utilizing our genetically encoded FRET-based biosensors, C kinase activity reporter 2 (CKAR2) and B kinase activity reporter (BKAR), to measure PKC and Akt activity, respectively, we demonstrate that both mutants are catalytically inactive in all kinases analyzed. Furthermore, the PKC mutants fail to autophosphorylate at the hydrophobic motif and thus are unable to adopt the autoinhibited conformation, resulting in accelerated membrane translocation in response to phorbol esters. This contrasts with β3-strand and R-spine PKCβII mutants, L373F and L394F, respectively, which retain activity, are phosphorylated at all three priming sites, and have normal autoinhibition. These findings highlight the value of C-spine mutations for generating catalytically inactive kinases that preserve the structural integrity of the kinase domain. Given that C-spine assembly is essential for the activity of every AGC kinase, these mutants offer broad applicability as a way to inactivate catalysis without impairing the kinase fold.

## Results

### Conserved residues in kinase domain mediate ATP binding and alignment of hydrophobic spines

**Figure 1:**
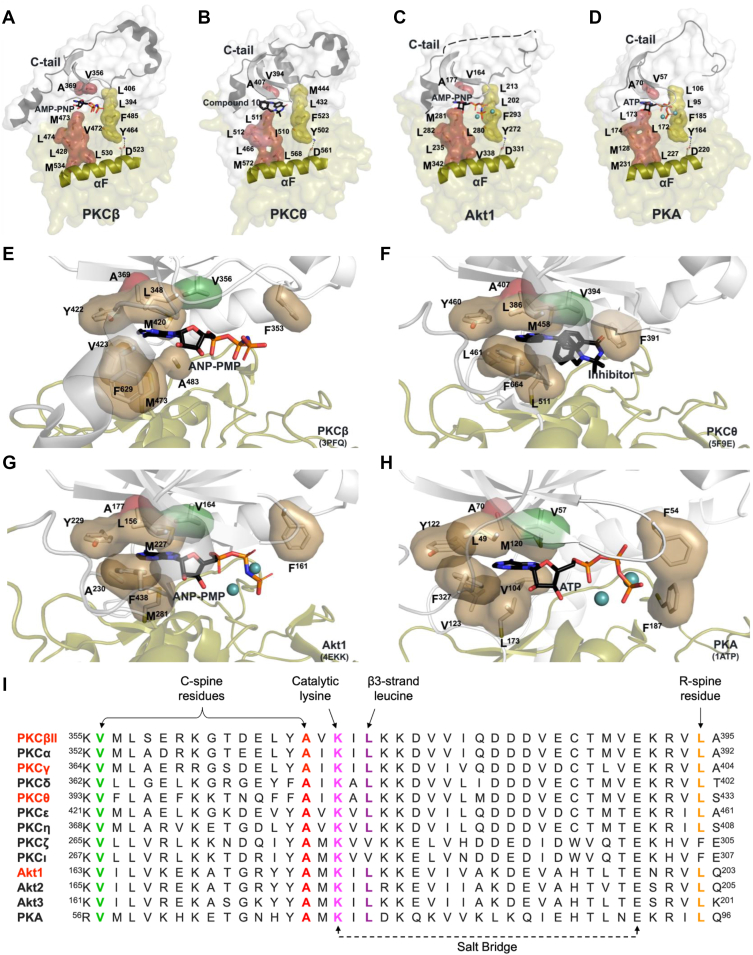
**Hydrophobic spine assembly by conserved residues in the kinase domain positions the enzyme for catalysis.***A*–*D*, kinase domain of PKCβ (PDB: 3PFQ; rat) *A*, PKCθ (PDB: 5F9E; human) *B*, Akt1 (PDB: 4EKK; human) *C*, and PKA (PDB: 1ATP; mouse) *D*, showing assembly of the catalytic (C) spine (*red*) and regulatory (R) spine (*yellow*) when bound to ATP, an ATP analog, or an ATP-competitive inhibitor. *E*–*H*, ATP-binding pocket of PKCβ *E*, PKCθ *F*, Akt1 *G*, and PKA *H*, highlighting residues involved in ATP coordination. The adenine ring of ATP is bound by C-spine residues valine and alanine in the N-lobe, and methionine or leucine in the C-lobe (V57, A70, and L173 in PKA). *I*, sequence alignment of PKC isozymes, Akt isozymes, and PKA showing conserved residues in the kinase domain. Kinases highlighted in *red* were analyzed in this study. C-spine residues, R-spine leucine (RS3), the invariant lysine, and the β3-strand leucine are indicated and analyzed in this study. Akt, protein kinase B; PDB, Protein Data Bank; PKA, protein kinase A; PKCβ, protein kinase C β; PKCθ, protein kinase C θ.

Hydrophobic spine assembly is a key feature of a closed, active kinase. ATP binding by conserved residues in the kinase domain brings the N- and C-lobes together, ensuring proper alignment of the two spines: the C-spine, which poises the kinase for catalysis, and the R-spine, which stabilizes the active conformation (46). Structural modeling of PKCβII, PKCθ, Akt1, and PKA bound to ATP, the nonhydrolyzable analog AMP-PNP, or inhibitor, demonstrates the assembly of the C-spine (*red*) and R-spine (*yellow*) (Fig. 1, *A*–*D*). Note, PKCγ lacks a crystal structure and was not included in the modeling. The R-spine consists of four residues, two from each lobe, whereas the C-spine is composed of eight residues, six from the C-lobe and two from the N-lobe (Fig. 1, *A*–*D*). ATP binding is mediated by C-spine residues valine and alanine in the N-lobe and methionine or leucine in the C-lobe (V356, A369, and M473 in PKCβII) (Fig. 1, *E*–*H*). Based on our modeling, the R groups of V356 and A369 in the C-spine are positioned toward the ATP-binding cavity of the kinase domain. Thus, we predicted that substitution with phenylalanine, which contains an aromatic, hydrophobic R group, would serve as a surrogate for ATP binding. Specifically, the aromatic ring at position 356 would partially fill the space occupied by the ribose ring of ATP, while the aromatic ring at position 369 would compete for the space occupied by the adenine ring of ATP. In this manner, the A369F and V356F mutations would facilitate spine assembly, preserving the scaffold function of the kinases. In addition, we predicted that mutation of these residues would impair ATP binding rendering the kinase catalytically inactive. Sequence analysis reveals these residues are highly invariant in the PKC, Akt, and PKA families (Fig. 1*I*; *green* and *red*). Indeed, A70 and V57 in PKA are as conserved as K72, E91, and D184 across the kinome. Interactions between kinase domain residues, including the catalytic lysine, R-spine residues, and β3-strand leucine, influence spine assembly and folding (15), and we hypothesize that the A70F and V57F also facilitate correct folding in the absence of ATP.

### PKCβII C-spine mutants are catalytically inactive and unphosphorylated

**Figure 2:**
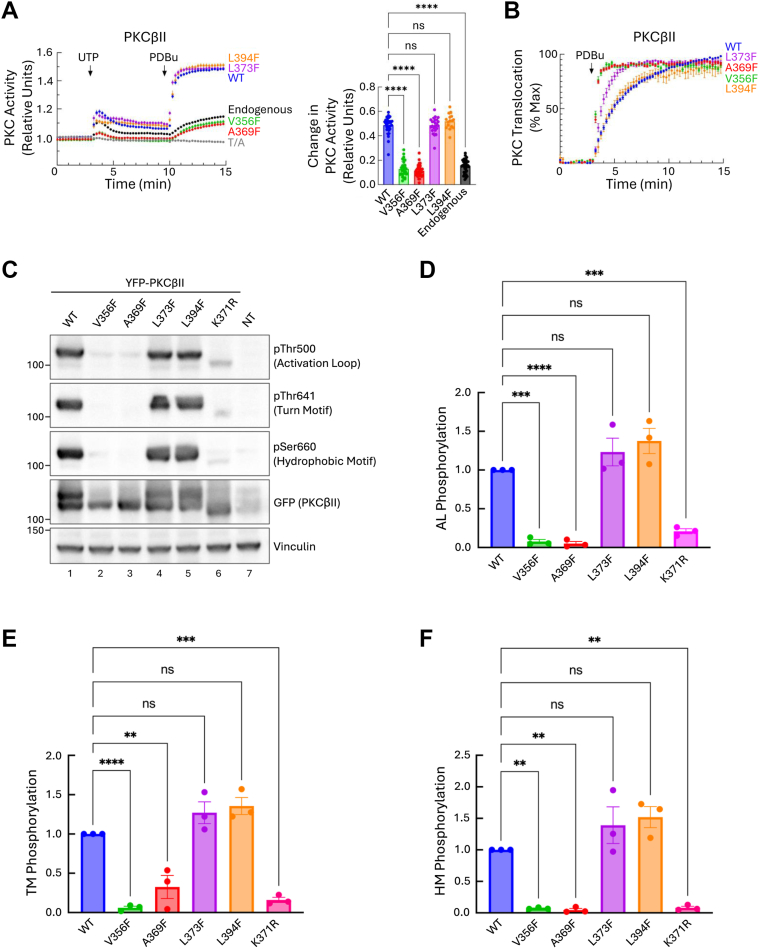
**C-spine, not R-spine, phenylalanine substitution in PKCβII renders the kinase inactive and unphosphorylated.***A*, *left*, PKC activity in COS7 cells expressing CKAR2 alone (*black trace*) or coexpressing CKAR2 and the indicated mCherry-tagged PKCβII constructs. At 3 min, cells were treated with 100 μM UTP followed by treatment with 200 nM PDBu at 9.7 min. Changes in FRET/CFP ratios were normalized to the first 3 min and plotted. Data are representative of 24 to 38 cells per condition from three independent experiments (mean ± SEM). A nonphosphorylatable reporter (CKAR2 TA; *gray trace*) was used as a control for any changes independent of PKC activity. *Right*, change in PKC activity measured as ΔFRET/CFP ratios between FRET/CFP ratios at 3 min and 15 min. Data were plotted and represent mean ± SEM. ns = nonsignificant, ∗∗∗∗*p* < 0.0001 by one-way ANOVA. *B*, PKC translocation in COS7 cells coexpressing MyrPalm-CFP and the indicated YFP-tagged PKCβII constructs treated with 200 nM PDBu at the indicated time. Changes in FRET/CFP ratios were normalized to the first 3 min and plotted as a percentage of max translocation. Data are representative of 21 to 43 cells per condition from three to five independent experiments (mean ± SEM). *C*, representative western blot of whole cell lysate from untransfected COS7 cells or COS7 cells expressing the indicated YFP-tagged PKCβII. *D*–*F*, quantification of PKCβII phosphorylation from *panel C* at the activation loop *D*, turn motif *E*, and hydrophobic motif *F*, normalized first to loading control then to total PKCβII protein. Data were plotted relative to the phosphorylation of PKCβII WT and represent mean ± SEM from three independent experiments. ns = not significant, ∗∗*p* < 0.01, ∗∗∗*p* < 0.001, and ∗∗∗∗*p* < 0.0001 by one-way ANOVA. CFP, cyan fluorescent protein; CKAR2, C kinase activity reporter 2; PDBu, phorbol 12, 13-dibutyrate; PKCβII, protein kinase C βII; YFP, *yellow fluorescent* protein.

To investigate the effect of the phenylalanine substitutions at key C-spine residues, we introduced each mutation individually into PKCβII, a conventional PKC isozyme extensively studied and used as a model for the PKC family (Fig. 2). We first measured the activity of the two mutants, V356F and A369F, using our genetically encoded FRET-based biosensor, CKAR2 (47, 48). Live-cell imaging with activity biosensors is an effective, fast, and highly reproducible way to measure the effects of mutations on the intrinsic catalytic activity of PKC and to reveal whether mutations are dominant negative (49, 50, 51). COS7 cells expressing CKAR2 alone (to assess endogenous activity, black trace) or with the relevant mCherry-tagged PKC were treated with uridine-5′-triphosphate (UTP), which activates purinergic receptors to produce DG and Ca^2+^ and transiently activate PKC (52), followed by treatment with phorbol 12,13-dibutyrate (PDBu), a PKC agonist, to maximally activate PKC (53). Treatment of cells expressing PKCβII WT with UTP resulted in a spike in activity before gradually decreasing, reflecting the metabolism of second messengers, DG and Ca^2+^. This was followed by a robust increase in activity upon treatment with PDBu (Fig. 2*A*, *blue trace*). Endogenous PKC activity exhibited a similar albeit milder response compared to overexpressed WT protein (Fig. 2*A*, *black trace*). Given the low expression of endogenous PKC in COS7 cells, this system provides an appropriate model for studying the effects of overexpressed constructs with minimal contribution from endogenous protein. The C-spine mutants, V356F and A369F, exhibited a significantly dampened response to both UTP and PDBu compared to wild-type PKC (Fig. 2*A*, *green* and *red traces*, respectively). This indicates that these mutants are catalytically inactive, with the observed FRET changes attributable to endogenous activity, as several PKC isozymes are expressed at low levels in COS7 cells, notably PKCα and PKCδ (54). Moreover, these mutants also act in a dominant negative manner toward endogenous PKC as evidenced by reduced amplitude of the activity curves. We also analyzed the effect of phenylalanine substitutions at L373 in the β3-strand and L394 in the R-spine, two residues predicted to stabilize the active conformation, and not inactivate, the kinase domain. Unlike C-spine mutations, Phe mutations of these R-spine residues facilitated assembly of the R-spine and were thus activating in a study of two other protein kinases (55). Similarly, we demonstrated that L373F and L394F in PKCβII were catalytically active and displayed a response similar to that of WT (Fig. 2*A*, *purple* and *orange traces*, respectively). These data indicate that mutations in the C-spine, and not the R-spine, abolish PKC catalytic activity.

We next examined the rate at which the spine mutants translocate to the plasma membrane in response to phorbol ester treatment. This informs on the degree of autoinhibition, as PKCs in a more open conformation translocate more rapidly due to increased exposure of the membrane-targeting domains (24). To assess the effect of the spine mutants on the autoinhibition of PKC, we performed a FRET-based translocation assay utilizing a plasma membrane-targeted cyan fluorescent protein (CFP) construct (MyrPalm-CFP) (56, 57). COS7 cells co-overexpressing MyrPalm-CFP with yellow fluorescent protein (YFP)-tagged PKCβII WT, V356F, A369F, L373F, or L394F were treated with PDBu and the resulting increase in FRET was measured as a readout for membrane association. In response to PDBu, PKCβII WT translocated to membranes with a half time of 2.3 ± 0.1 min (Fig. 2*B*, *blue trace*). This rate is consistent with the agonist-dependent membrane translocation kinetics of a properly folded PKCβII (58). In contrast, the two C-spine mutants, V356F and A369F, translocated to the membrane at a 5-fold faster rate compared to WT (Fig. 2*B*, t_1/2_ = 0.38 ± 0.03 min for V356F (*green*) and 0.43 ± 0.02 min for A369F (*red*), respectively). This indicates that autoinhibition is reduced in these mutants. The R-spine mutant, L394F, translocated to the membrane at a similar rate to WT, indicative of a properly autoinhibited kinase (Fig. 2*B*, t_1/2_ = 1.8 ± 0.1 min, *orange trace*). Interestingly, the β3-strand mutant, L373F, translocated to the membrane slightly faster than WT (Fig. 2*B*, t_1/2_ = 1.1 ± 0.1 min, *purple trace*). This suggests that although the L373F mutant maintains kinase activity, the phenylalanine substitution, by strengthening the R-spine, may slightly disrupt autoinhibition. Taken together, these data reveal that C-spine mutants adopt a more open conformation with their membrane-targeting moieties exposed, resulting in faster membrane translocation.

Given that PKC mutants with reduced autoinhibition are more susceptible to dephosphorylation by phosphatases (59), we next examined phosphorylation of the spine mutants by western blot. Unstimulated COS7 cells expressing YFP-tagged PKCβII WT, V356F, A369F, L373F, or L394F were lysed and probed for phosphorylation at the activation loop, turn motif, and hydrophobic motif. We also analyzed the PKCβII mutant, K371R, in which an active-site substitution renders PKC catalytically inactive, as well as nontransfected cells serving as a negative control. PKCβII WT, L373F, and L394F were phosphorylated at all three priming sites, indicating normal processing (Fig. 2*B*, lanes 1, 4, and 5; Fig. 2, *D*–*F*). In contrast, phosphorylation was nearly undetectable in the two C-spine mutants, V356F and A369F, as well as the K371R mutant (Fig. 2*C*, lanes 2, 3, and 6; Fig. 2, *D*–*F*). No immunoreactivity was detected in control lysate from nontransfected cells (Fig. 2*C*, lane 7). These data are consistent with our imaging analyses showing that spine mutants are (1) inactive, and therefore cannot autophosphorylate on the hydrophobic motif, and (2) in an open conformation and thus unable to retain phosphate on the turn motif and activation loop sites.

### C-spine phenylalanine substitutions in another cPKC, PKCγ, also render the kinase inactive and unphosphorylated

**Figure 3:**
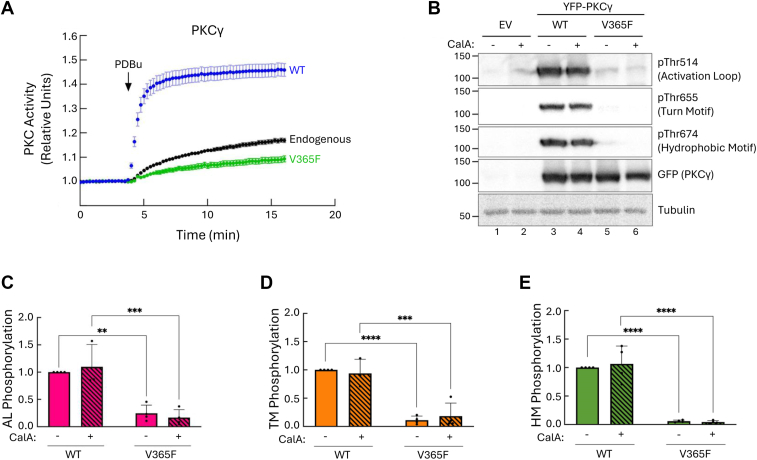
**PKCγ C-spine phenylalanine substitution, V365F, is also inactive and unphosphorylated.***A*, PKC activity in COS7 cells expressing CKAR2 alone (*black trace*) or coexpressing CKAR2 and mCherry-PKCγ WT or V365F. At 3 min, cells were treated with 200 nM PDBu. Changes in FRET/CFP ratios were normalized to the first 3 min and plotted. Data are representative of 15 to 20 cells per condition from three independent experiments (mean ± SEM). *B*, representative western blot of whole cell lysate from COS7 cells expressing YFP-empty vector or the indicated YFP-tagged PKCγ constructs. Cells were treated with 50 nM CalA or DMSO control for 10 min. *C*–*E*, quantification of PKCγ phosphorylation from panel B at the activation loop *C*, turn motif *D*, and hydrophobic motif *E*, normalized to total PKCγ protein and loading control. Data were normalized to the phosphorylation of DMSO-treated PKCγ WT and represent mean ± SEM from four independent experiments. ∗∗*p* < 0.01, ∗∗∗*p* < 0.001, and ∗∗∗∗*p* < 0.0001 by two-way ANOVA and Tukey *post hoc* test. Relevant statistical comparisons are shown. CalA, calyculin A; CKAR2, C kinase activity reporter 2; DMSO, dimethyl sulfoxide; PDBu, phorbol 12, 13-dibutyrate; PKCγ, protein kinase C γ; YFP, yellow fluorescent protein.

We next asked whether the equivalent phenylalanine substitution in PKCγ, a conventional PKC isozyme highly expressed in neurons (60), also results in a catalytically inactive kinase. We first assessed the activity of the mutant, V365F, using CKAR2. COS7 cells expressing CKAR2 alone, or coexpressing mCherry-tagged PKCγ WT or V365F were treated with PDBu. As observed with PKCβII, treatment of cells expressing PKCγ WT with PDBu resulted in a robust increase in activity (Fig. 3*A*, *blue trace*). However, the V365F mutant exhibited a small increase in activity that was lower than endogenous PKC, indicating that the mutant is catalytically inactive and dominant negative towards the endogenous PKC (Fig. 3*A*, *green* and *black traces*, respectively).

We next assessed whether the PKCγ V365F mutant also was unable to autophosphorylate at the hydrophobic motif and retain phosphate at the activation loop and turn motif. COS7 cells expressing YFP-tagged PKCγ WT or V365F were lysed, and phosphorylation was assessed with phospho-specific antibodies. PKCγ WT was phosphorylated at all three priming sites, and no increase was observed in the presence of the phosphatase inhibitor calyculin A (CalA) (Fig. 3*B*, lanes 3 and 4; Fig. 3, *C*–*E*). In contrast, the V365F mutant showed no phosphorylation at the hydrophobic or turn motifs, and only minimal phosphorylation at the activation loop (Fig. 3*B*, lanes 5 and 6; Fig. 3, *C*–*E*). Inhibition of phosphatases with CalA did not promote phosphorylation. The lack of phosphorylation of the V365F mutant suggests that PKC is unable to fully mature and autoinhibit. These results show that, as with PKCβII, substitution of valine to a phenylalanine in the C-spine of PKCγ not only inactivates the kinase but also prevents autoinhibition and retention of phosphate at the processing sites.

### C-spine mutations in the novel PKCθ abolish its activity and phosphorylation without affecting stability

**Figure 4:**
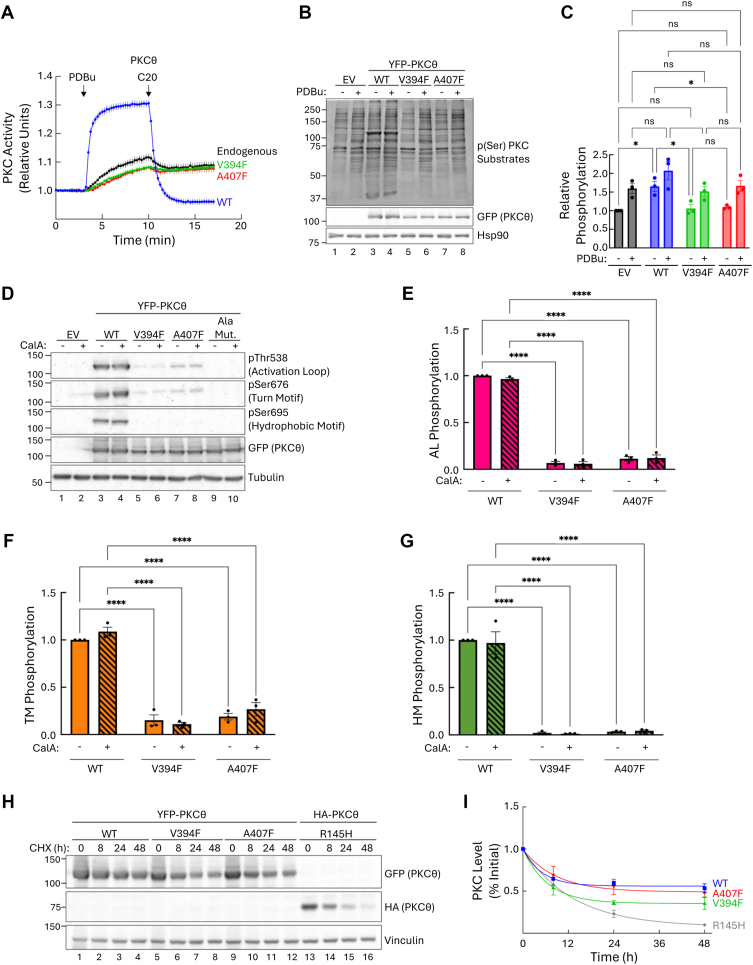
**PKCθ C-spine mutants are inactive and unphosphorylated but remain stable.***A*, PKC activity in COS7 cells expressing CKAR2 alone (*black trace*) or co-expressing CKAR2 and mCherry-PKCθ WT, V394F, or A407F. At 3 min, cells were treated with 200 nM PDBu followed by 1 μM of the PKCθ-specific inhibitor, C20, at 10 min. Changes in FRET/CFP ratios were normalized to the first 3 min and plotted. Data are representative of 33 to 50 cells per condition from three independent experiments (mean ± SEM). *B*, representative western blot of whole cell lysate from COS7 cells transfected with YFP-empty vector (EV) or the indicated YFP-tagged PKCθ constructs and treated with 200 nM PDBu or DMSO control for 15 min. PKCθ activity was assessed by probing for phospho-Ser PKC substrates. *C*, phospho-Ser PKC substrate signal from panel B was quantified by densitometry of the full lane excluding the 100 kDa band corresponding to PKCθ. Data were normalized first to loading control then to total PKCθ protein and plotted relative to untreated EV. Data represent mean ± SEM from three independent experiments. ns = not significant, ∗*p* < 0.05 by two-way ANOVA and Tukey *post hoc* test. Relevant statistical comparisons are shown. *D*, representative western blot of whole cell lysate from COS7 cells expressing YFP-EV or the indicated YFP-tagged PKCθ constructs. Corresponding phosphorylation site alanine mutants were included as a control for antibody specificity. Cells were treated with 50 nM CalA or DMSO control for 10 min. *E*–*G*, quantification of PKCθ phosphorylation from *panel D* at the activation loop *E*, turn motif *F*, and hydrophobic motif *G*, normalized first to loading control then to total PKCθ protein. Data were plotted relative to the phosphorylation of DMSO-treated PKCθ WT and represent mean ± SEM from three independent experiments. ∗∗∗∗*p* < 0.0001 by two-way ANOVA and Tukey *post hoc* test. Relevant statistical comparisons are shown. *H*, representative western blot of whole cell lysate from COS7 cells expressing the indicated YFP-tagged PKCθ constructs or HA-tagged PKCθ R145H; note the nature of the tag does not alter stability as HA-tagged PKCθ WT has a half-life of 37 ± 5 min (63) comparable to that of YFP-tagged PKCθ WT reported here (39 ± 4 min). Cells were treated with 355 μM CHX and lysed at the indicated times. *I*, quantification of PKCθ protein levels from *panel H* normalized to loading control and plotted relative to the 0 h time point. Data were fit to a first-order decay and depict mean ± SEM from three independent experiments. C20, compound 20; CalA, calyculin A; CHX, cycloheximide; CKAR2, C kinase activity reporter 2; DMSO, dimethyl sulfoxide; HA, hemagglutinin; PDBu, phorbol 12, 13-dibutyrate; PKCθ, protein kinase C θ; YFP, *yellow* fluorescent protein.

Once we established that C-spine mutations in conventional PKCs led to loss of phosphorylation and catalytic activity, we sought to determine if the same was the case for novel PKC isozymes. Using PKCθ as our representative novel PKC, we measured the activity of the two mutants (V394F and A407F in PKCθ) in live cells using our PKC activity reporter, CKAR2. We co-overexpressed mCherry-tagged PKCθ WT, V394F, or A407F with CKAR2 in COS7 cells and treated with PDBu followed by the PKCθ-specific inhibitor, compound 20 (C20) (61). We additionally measured the activity of endogenously expressed PKCs by transfecting with CKAR2 alone. Treating cells expressing PKCθ WT with PDBu resulted in maximal activity which was reversed upon treatment with C20 (Fig. 4*A*, *blue trace*). Endogenous PKC activity showed a modest increase in activity that was largely unaffected by C20 treatment; the slight dip in activity can be attributed to endogenous PKCδ which is expressed in COS7 cells and sensitive to this inhibitor, whereas PKCθ is not expressed in this system (Fig. 4*A*, *black trace*) (62, 63). The two mutants, V394F and A407F, displayed a lower response to that of endogenous PKCs, indicating that they are catalytically dead and dominant negative toward endogenous PKC (Fig. 4*A*, *green* and *red traces*, respectively).

To corroborate our live-cell imaging results revealing that the spine mutants are inactive, we assessed activity of the mutants by quantifying phosphorylation of PKC substrates by western blot. COS7 cells expressing YFP-tagged PKCθ WT, V394F, or A407F were treated with PDBu for 15 min, and whole-cell lysates were probed using a phospho-Ser PKC substrate antibody. Cells expressing YFP empty vector (EV) were used as a negative control. In unstimulated cells expressing EV, we observed basal phosphorylation of PKC substrates, which significantly increased with PDBu treatment (Fig. 4*B*, lanes 1 and 2; Fig. 4*C*). Unstimulated cells expressing PKCθ WT displayed increased substrate phosphorylation compared to EV, reflecting the elevated basal activity we have previously reported for this isozyme (63). A significant increase in phosphorylation was observed with PDBu treatment (Fig. 4*B*, lanes 3 and 4; Fig. 4*C*). The two mutants, V394F and A407F, showed a significant reduction in substrate phosphorylation in both untreated and treated conditions compared to WT, but were indistinguishable from EV (Fig. 4*B*, lanes 5–8; Fig. 4*C*). These results are consistent with our imaging analysis that the two C-spine mutants are kinase-dead. Note the dominant negative effect was not observed in this assay because of the lower sensitivity of western blot detection compared to the CKAR2 assay.

Next, we assessed phosphorylation of the PKCθ C-spine mutants using western blot. COS7 cells expressing YFP-tagged PKCθ WT, V394F, or A407F were treated with the phosphatase inhibitor, CalA, to prevent dephosphorylation, and whole-cell lysates were probed for phosphorylation at the activation loop, turn motif, and hydrophobic motif. The corresponding alanine mutant at each phosphorylation site served as a negative control for antibody specificity, and YFP EV was used as a negative control for background binding. As expected, PKCθ WT was phosphorylated at all three sites, reflecting the constitutive phosphorylation typical of PKC isozymes (Fig. 4*D*, lanes 3 and 4; Fig. 4, *E*–*G*). In contrast, both V394F and A407F had significantly impaired phosphorylation at all three sites (Fig. 4*D*, lanes 5–8; Fig. 4, *E*–*G*). No differences were observed with CalA treatment for any of the constructs.

Typically, PKC isozymes that do not undergo processing by phosphorylation at the priming sites are unstable and rapidly degraded (26). Therefore, we next asked whether the C-spine mutants, which lack these phosphorylations, were turned over more rapidly. To elucidate this, we analyzed the half-time for degradation of the V394F and A407F mutants in unstimulated cells. COS7 cells overexpressing YFP-tagged PKCθ WT, V394F, or A407F were treated with cycloheximide (CHX) for up to 48 h to inhibit protein synthesis, and total PKCθ protein levels were quantified by western blot. As a positive control, we overexpressed the PKCθ cancer-associated mutant, R145H, which harbors a histidine substitution at position 145 in the pseudosubstrate. We have previously shown that this mutant is unstable and rapidly degraded (Fig. 4*H*, lanes 13–16; Fig. 4*I*, gray trace) (63). PKCθ WT was stable and had a half-life of 37 ± 5 h (Fig. 4*H*, lanes 1–4; Fig. 4*I*, *blue trace*). Surprisingly, the two C-spine mutants, V394F and A407F were also stable, and their degradation rate did not differ significantly from WT (Fig. 4*H*, lanes 5–12; Fig. 4*I*, *green* and *red traces*, respectively). These data reveal that even without the priming phosphorylations, the C-spine mutants retain their stability.

### C-spine mutations in Akt render the kinase inactive with decreased phosphorylation at the hydrophobic motif

**Figure 5:**
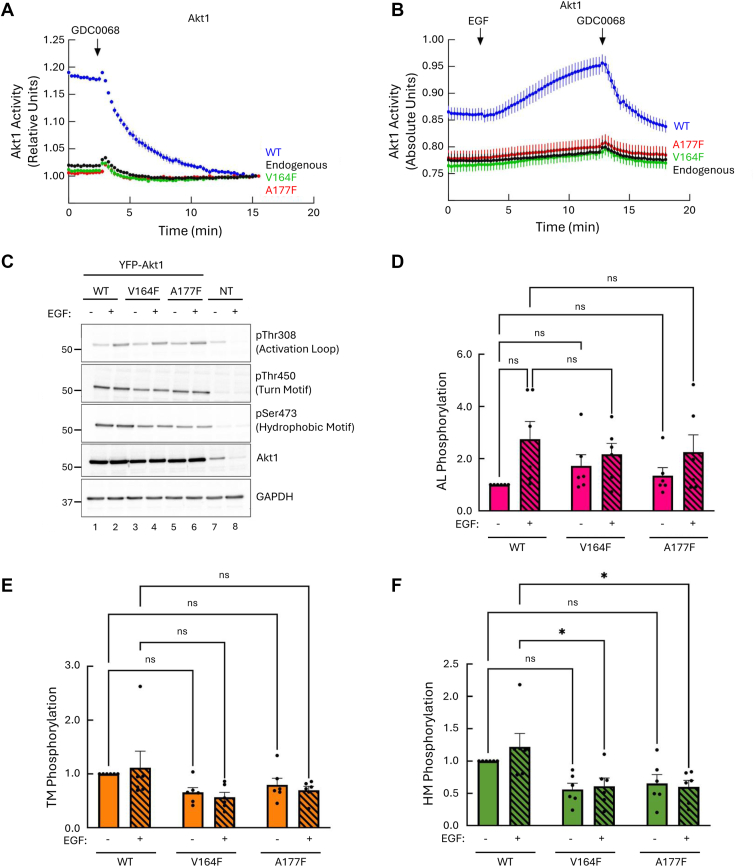
**Akt1 C-spine mutants are inactive with reduced phosphorylation at the hydrophobic motif.***A*–*B*, Akt activity in COS7 cells expressing BKAR and empty vector (*black trace*) or coexpressing BKAR and mCherry-Akt1 WT, V164F, or A177F. Cells grown in 10% serum *A* or serum starved for 18 h before imaging *B*. At 3 min, cells were treated with *A*, 20 μM of the Akt inhibitor, GDC0068 alone, or *B*, 50 ng/ml EGF followed by 20 μM of GDC0068 at 10 min. Changes in FRET/CFP ratios were normalized to the first 3 min *A* and plotted or absolute changes in FRET/CFP ratios were plotted *B*. Data are representative of 36 to 46 cells per condition from three to four independent experiments (mean ± SEM). *C*, representative western blot of whole cell lysate from COS7 cells transfected with the indicated HA-tagged Akt1 constructs. Cells were serum starved for 15 h before treating with DMSO or 50 ng/ml EGF for 8 min. *D*–*F*, quantification of Akt1 at the activation loop *D*, turn motif *E*, and hydrophobic motif *F*, from panel C normalized to loading control and total Akt1 protein. Data were normalized to the phosphorylation of DMSO-treated Akt1 WT and represent mean ± SEM from six independent experiments. ns = nonsignificant, ∗*p* < 0.05, ∗∗*p* < 0.01, and ∗∗∗*p* < 0.001 by two-way ANOVA and Tukey *post hoc* test. Relevant statistical comparisons are shown. Akt, protein kinase B; BKAR, B kinase activity reporter; CFP, cyan fluorescent protein; DMSO, dimethyl sulfoxide; EGF, epidermal growth factor; HA, hemagglutinin.

To investigate the applicability of the C-spine phenylalanine substitutions across the AGC kinase family, we next introduced these mutations into Akt1, a ubiquitously expressed isoform of the Akt kinase family. We assessed the activity of the mutants, V164F and A177F, using a genetically encoded FRET-based Akt activity reporter, BKAR (57). COS7 cells, maintained in serum-containing media, expressing BKAR alone (to measure endogenous Akt activity) or coexpressing mCherry-tagged Akt1 WT, V164F, or A177F were treated with GDC0068, an ATP-competitive inhibitor to inhibit Akt1 activity, and the reduction in FRET was used as a measure of the basal activity of Akt. In cells expressing only the reporter, inhibitor treatment resulted in a small drop in FRET, representing the low basal activity of endogenous Akt (Fig. 5*A*, *black trace*). In cells expressing Akt1 WT, inhibitor treatment resulted in a large drop in FRET, reflecting the basal activity of the overexpressed Akt1 (Fig. 5*A*, *blue trace*). In contrast, inhibitor addition to cells expressing the C-spine mutants, V164F or A177F, produced responses that were indistinguishable from cells with no overexpressed Akt, revealing no activity of the mutants (Fig. 5*A*, *green* and *red traces*, respectively). To assess whether activity could be induced by stimulating Akt, cells were serum starved and subsequently treated with epidermal growth factor (EGF) to produce PIP_3_, activating Akt (59), followed by GDC0068 to inhibit Akt1 activity. Treatment of cells expressing Akt1 WT with EGF resulted in a robust increase in Akt1 activity, suggesting that EGF stimulation maximized Akt1 activity as indicated by the increase in activity above basal levels. Following treatment with GDC0068, WT activity dropped below baseline, consistent with the high basal activity depicted in panel A (Fig. 5*B*, *blue trace*). Endogenous Akt1 also displayed a slight increase in activity following EGF stimulation that was reversed upon GDC0068 treatment (Fig. 5*B*, *black trace*). The two C-spine mutants, V164F and A177F, exhibited a similar increase in activity after EGF stimulation to endogenous Akt1, suggesting that the increase in activity results from endogenous Akt1 rather than the mutant Akt1 (Fig. 5*B*, *green* and *red traces*, respectively; note the inhibitor is unlikely to bind the spine mutants but they have no activity and thus is not relevant). Unlike with the PKC spine mutants, the Akt spine mutants had no effect on endogenous Akt activity and are thus not dominant negative. Taken together, this result shows that despite agonist stimulation to maximize Akt1 activity, both C-spine mutants remain catalytically inactive.

We next addressed how mutation of the spine residues affected the phosphorylation of Akt. Although Akt contains the same phosphorylation sites as PKC, the modifications are catalyzed at different times and have different functions compared with PKC. Notably, the turn motif is phosphorylated cotranslationally (and thus also constitutive, as for the PKC isozymes), whereas the activation loop and hydrophobic motif are phosphorylated only upon binding of the PH domain to PIP_3_, to expose the activation loop for phosphorylation by PDK1, in turn triggering autophosphorylation of the hydrophobic motif. Both sites are used as markers for the activation of Akt; however, the activation loop phosphorylation correlates best with activity and is a better prognostic marker in cancer (34, 64, 65, 66). COS7 cells expressing hemagglutinin (HA)-tagged Akt1 WT, V164F, or A177F were treated with dimethyl sulfoxide (DMSO) control or stimulated with EGF. Western blot analysis of lysates revealed that Akt1 WT was basally phosphorylated at the activation loop, turn motif, and hydrophobic motif (Fig. 5*C*, lane 1 and *D*–*F*) and that phosphorylation of the activation loop increased upon treatment of cells with EGF (Fig. 5*C*, lane 2; Fig. 5*D*) (22, 23, 24, 25). Interestingly, both V164F and A177F mutants were also phosphorylated at the activation loop and turn motif but exhibited a significant reduction in phosphorylation at the hydrophobic motif that was unaffected by EGF stimulation (Fig. 5*C*, lanes 3–6; Fig. 5, *D*–*F*). Unlike the activation loop and turn motif, whose phosphorylation is facilitated by PDK1 and mTORC2, respectively, phosphorylation at the hydrophobic motif occurs by autophosphorylation (25, 35, 36, 37). Thus, the reduced phosphorylation at the hydrophobic motif on the V164F and A177F mutants reflects the lack of catalytic activity of the enzyme. Note that unlike with PKC, where phosphorylation of the hydrophobic motif was abolished in C-spine mutants, approximately 50% phosphorylation remained on the hydrophobic motif of the Akt C-spine mutants. Whether this is a result of phosphorylation by endogenous Akt in COS7 cells or by a separate kinase remains to be determined. Importantly, unlike PKC, the impaired autophosphorylation of the hydrophobic motif had minimal effects on retention of phosphate at the other sites. Overall, these results show that phenylalanine substitution of key C-spine residues also renders Akt inactive.

## Discussion

**Figure 6:**
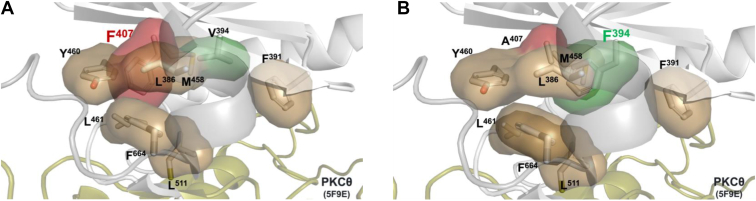
**Phenylalanine mutation at C-spine residues mimics ATP binding.***A*–*B*, ATP-binding pocket of PKCθ with A407 (*red*) *A*, or V394 (*green*) *B*, substituted with phenylalanine, illustrating how the phenyl group is a surrogate for the adenine ring of ATP to assemble the C-spine. PKCθ, protein kinase C θ.

Kinase-dead mutants have widespread use as a tool for studying protein kinase function, but commonly used mutants, which change residues that participate in catalysis, impair the fold of the kinase domain (67, 68, 69, 70, 71, 72, 73, 74, 75). Here, we take advantage of C-spine assembly to generate kinase-inactive mutants that maintain a proper active fold of the kinase domain. Specifically, we analyzed two residues whose mutation to phenylalanine abolished activity in three PKC isozymes (βII, γ, and θ) and Akt1. These residues, V394 and A407 in PKCθ, are located in the C-spine of the kinase domain, and mutation to a phenylalanine acts as a surrogate for the adenosine ring of ATP; they fuse the hydrophobic core architecture of the C-spine to create a stable and properly aligned kinase core (Fig. 6). Further analysis of these PKC mutants revealed that they are unphosphorylated at the activation loop, turn motif, and hydrophobic motif, leading to decreased autoinhibition. This is supported by our findings that PKCβII mutants translocate to the plasma membrane more rapidly than WT. Surprisingly, despite the mutants showing impaired autoinhibition, the PKCθ mutants remained stable overtime. A decrease in phosphorylation at the hydrophobic motif, but not the activation loop or turn motif, was observed for Akt1, consistent with this being a known autophosphorylation site. Analysis of two other mutants (L373F and L394F in PKCβII), located in the β3-strand and R-spine of the kinase core, respectively, revealed these to be active and phosphorylated at all three priming sites. Taken together, these data suggest that C-spine mutations, particularly those at residues involved in ATP binding, are superior for generating inactive protein kinases by creating stable pseudokinases.

Traditional kinase-dead mutants are generated by mutating the invariant catalytic lysine, which plays an essential role in the transfer of phosphate to protein substrates but does not directly bind ATP (76, 77). The lysine is often replaced with a positively charged arginine or a similarly sized methionine to mitigate structural changes (78, 79, 80, 81). Although such mutations typically abolish kinase activity, they may also destabilize the kinase domain or induce alternative conformations (7, 10, 82, 83). This raises the question of whether the loss of activity is primarily due to structural changes rather than the absence of ATP coordination. Moreover, several studies have shown that some lysine mutants retain kinase activity (10, 84, 85, 86). In one study of atypical PKCɩ, the authors found that mutation to arginine had no effect on catalytic activity, while mutation to tryptophan completely abolished activity (10). Thus, different substitutions at the same residue can have markedly different effects. The notion that lysine mutants can retain residual activity is further supported by pseudokinases. Although pseudokinases lack critical catalytic residues and were initially considered inactive, some, like KSR and HER3, have been shown to retain low levels of activity (14, 87). This suggests that mutating the invariant lysine may not always be sufficient to completely abolish kinase activity. Although our study did not examine the activity of invariant lysine mutants, we observed low levels of the priming phosphorylations in PKCβII K371R, which may indicate residual activity (Fig. 2*C*, lane 6; Fig. 2, *D*–*F*). Similarly, mutation of the catalytic Asp in PKCε (Asp532) was shown to still allow phosphorylation of the hydrophobic motif (88), with subsequent pulse-chase analyses showing that this mutation in PKCβII decreased the rate of processing and that a second weakening mutation (in this case replacement of the activation loop Thr with a weak phosphomimetic, Glu) was needed to abolish hydrophobic motif autophosphorylation (89). Our C-spine mutants thus serve as a particularly effective mutation to abolish ATP binding and catalysis.

Our generation of stable pseudokinases through mutation of C-spine residues mimics occurrences in nature. Across the kinome, pseudokinases frequently arise from mutations in highly conserved motifs required for catalysis, yet they often maintain structural features that enable noncatalytic roles. For example, the Ser/Thr family member VRK3 lacks enzymatic activity due to mutations in the ATP-binding pocket, but it preserves the conformation of its active paralog, VRK2, thereby retaining scaffolding function to regulate Erk signaling (90). Similarly, receptor tyrosine kinase pseudokinases such as RYK, ROR2, and PTK7 contribute to Wnt signaling despite lacking catalytic activity, highlighting the capacity of pseudokinases to act as signaling scaffolds or modulators (91). In the nonreceptor tyrosine kinase family, the pseudokinase MLKL demonstrates an alternative adaptation: although catalytically inactive, it is activated by upstream phosphorylation that causes conformational changes to drive necroptosis (92). Furthermore, nature has also taken advantage of C-spine mutations in disease. ProKinO, an ontology tool for searching cancer kinome databases, has also identified various cancer-associated C-spine mutations, such as a valine to phenylalanine substitution in the receptor tyrosine kinase ErbB4 (V732F) and in the serine/threonine kinase BRAF (V471F) in lung carcinoma (93). Collectively, these examples illustrate how pseudokinases can evolve to lose enzymatic function while retaining or even acquiring specialized regulatory roles in signaling pathways.

The highly conserved kinase domain of protein kinases contains two essential structural elements: the regulatory (R) spine and the catalytic (C) spine, known as the hydrophobic spines (14, 17, 46). These spines are a hallmark of all kinases, contributing to enzymatic function and stability (46). The R-spine consists of four residues, RS1 to RS4, that must be assembled to form the active conformation of the kinase (14). Disassembly of the R-spine disrupts this conformation, shifting the kinase to an inactive state, functioning as a switch to regulate activity (14). Each of these residues is highly conserved and located within key structural motifs: RS1 is the histidine/tyrosine from the HRD motif, RS2 is the phenylalanine from the DFG motif, RS3 is an aliphatic residue in the αC-helix, and RS4 is an aliphatic residue in the β4-strand (Y164, F185, L95, and L106 in PKA, respectively) (46, 55). The C-spine consists of several hydrophobic residues spanning both the N- and C-lobes and is assembled upon ATP binding (17). ATP binding brings the two lobes together, positioning the kinase for catalysis (17). Studies have identified the valine and alanine in the N-lobe, as well as the leucine or methionine in the C-lobe, as critical residues for mediating interactions with the adenine ring of ATP (94).

Several studies have shown that mutations in the R- or C-spine can modulate kinase activity. Hu et al. first demonstrated that substituting a conserved C-spine alanine in KSR1 with phenylalanine, but not valine, blocked ATP binding and inactivated the kinase while still permitting scaffolding through CRAF binding (19). Similar results were observed with equivalent mutations in CRAF and BRAF, with modeling suggesting that phenylalanine mimicked ATP binding to stabilize C-spine assembly (18). In contrast, R-spine mutations such as RS3 leucine to phenylalanine rendered CRAF and BRAF constitutively active, consistent with enforced R-spine assembly (95). Importantly, cancer genomics databases revealed recurrent phenylalanine substitutions at key residues, including a valine in the C-spine and a leucine in the β3-strand, underscoring that these alterations also occur in nature (55). As in the earlier studies, the valine mutation resulted in inactivity, while the leucine mutation preserved activity. For C-spine residues, only substitution with phenylalanine effectively blocked ATP binding and induced the active-like conformation (55). The above studies highlight that while both spines are essential for kinase activity, mutations in these residues lead to distinct outcomes: C-spine mutations generate inactive kinases, whereas R-spine mutations lead to constitutively active kinases. Crucially, proper assembly of both spines is maintained, and C-spine mutations can be used to differentiate between kinase and scaffolding functions.

In this study, we focus on the C-spine residues valine and alanine in PKC isozymes (βII, γ, and θ) and Akt1. These C-spine residues are highly conserved across the AGC family of kinases (Fig. S1) (96). In PKC, phenylalanine mutations rendered the kinases inactive and prevented phosphorylation at the activation loop, turn motif, and hydrophobic motif. The absence of phosphorylation at the hydrophobic motif, a known autophosphorylation site, can be attributed to the inactivity of the mutants, whereas the absence of phosphorylation at the activation loop and turn motif can be attributed to the activity of protein phosphatases. PKC that lacks phosphorylation at the hydrophobic motif is unable to adopt an autoinhibited conformation, which causes a two orders of magnitude increase in the sensitivity of PKC to dephosphorylation (97), triggering dephosphorylation of the activation loop and turn motif by protein phosphatase 1 (PP1) and 2A (PP2A) (98, 99). Conversely, the two Akt1 C-spine mutants had reduced hydrophobic motif phosphorylation but retained phosphorylation at the activation loop and turn motif. Since Akt phosphorylation regulates activity rather than stability, only the hydrophobic motif, an autophosphorylation site, was affected. These findings align with the established regulatory mechanisms of Akt (31).

Typically, PKC that is unable to autoinhibit is rapidly dephosphorylated, ubiquitinated, and degraded by the proteasome (100). However, our data reveal that the C-spine mutants in PKCθ are not rapidly degraded despite their lack of phosphorylation, whereas R145H, an unphosphorylated cancer-associated mutant (63), is rapidly degraded. Gould et al. have previously reported active site inhibitors to stabilize PKC by preventing the mature enzyme from dephosphorylation (89). Whereas the C-spine mutants lack phosphorylation, the phenylalanine substitution blocks ATP binding by occupying the active site of the kinase domain, mimicking the effects of many ATP-competitive inhibitors. Active site occupancy by the pseudosubstrate, peptide substrates, or ATP preventing the dephosphorylation of PKC has also been reported (99, 101, 102), suggesting that the phenylalanine occupying the active site may play a role in the increased stability of the C-spine mutants. However, how the occupancy increases stability despite the lack of phosphorylation at the activation loop, turn motif, and hydrophobic motif remains to be determined.

For PKC, the C-spine mutants were not only inactive, but also exhibited a dominant negative effect by decreasing endogenous PKC activity. Previous studies have reported other kinase-dead PKC mutants to act in a dominant negative manner, particularly loss-of-function cancer mutants (10, 49, 103). One possible mechanism for this effect is that mutant PKC impairs the phosphorylation of other PKCs leading to their degradation (104). In this regard, our lab recently demonstrated that nascent, unphosphorylated PKC forms a homodimer mediated by the C-tail, which is prone to degradation if not properly processed (25). Thus, mutant unphosphorylated PKC likely dimerizes with wild-type PKC, resulting in the dominant negative effect in part from preventing processing and also by simply allosterically inhibiting the WT enzyme. Our data also showed that the mutant PKC retains the ability to translocate to the plasma membrane, suggesting that the C1 and C2 domains respond to agonist stimulation and may cause a dominant negative effect by sequestering second messengers, DG and Ca^2+^. In contrast to PKC, the Akt1 C-spine mutants, although inactive, did not act in a dominant negative manner. Unlike PKC, Akt has not been reported to dimerize, suggesting that the dominant negative effect is more likely a result of dimerization rather than impaired phosphorylation or sequestration of second messengers.

We also analyzed two leucine mutants in the β3-strand and R-spine of PKCβII, L373F, and L394F, respectively. L394 is RS3 in the R-spine, while L373 corresponds to L74 in PKA which was previously identified using cancer genomics databases as a residue that influences R-spine assembly (55). L373 is located in the loop between the β3-strand and the αC-helix and is predicted to stabilize the αC-helix through interactions with nearby residues (55). Analysis of these mutants revealed that they are indistinguishable from wild-type in both activity and phosphorylation state. Unlike CRAF and BRAF where the corresponding mutations render the kinases constitutively active, the PKCβII R-spine mutants are not constitutively active and exhibit similar activation and inactivation kinetics as the WT enzyme. However, a methionine mutation at the equivalent residue in CRAF and BRAF was more effective at generating a constitutively active kinase, indicating that different residues may be better or worse at stabilizing the R-spine (55).

**Figure 7:**
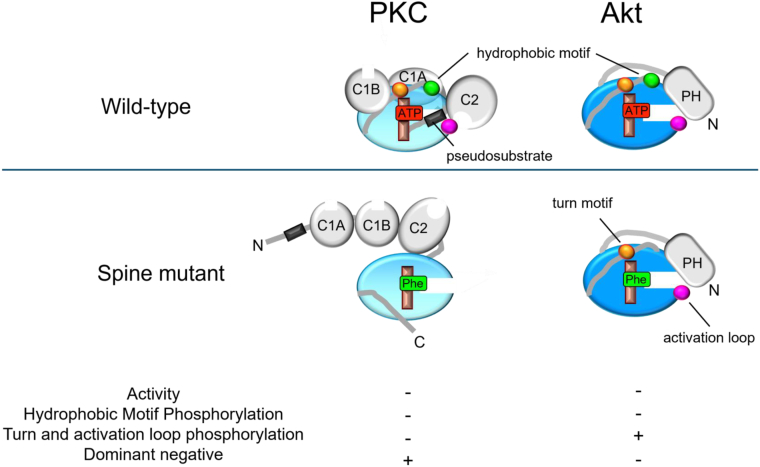
**C-spine mutations inactivate both PKC and Akt but with different outcomes on their phosphorylation states.***Top panel*: in wild-type PKC and Akt, the adenosine ring of ATP (*red*) assembles the C-spine (*pale red*), permitting processing phosphorylations. For PKC, these are the mTORC2-catalyzed phosphorylation at the turn motif (*orange circle*), the PDK1-catalyzed phosphorylation of the activation loop (*magenta circle*), and the autophosphorylation of the hydrophobic motif (*green*); phosphorylation of the hydrophobic motif is necessary for PKC to adopt the autoinhibited conformation with the pseudosubstrate (*dark gray*) in the substrate-binding cavity. Acute activity is controlled by second messenger binding to relevant domains. Akt undergoes the same phosphorylations but differs in that the phosphorylation by PDK1 at the activation loop (*magenta circle*) and subsequent autophosphorylation at the hydrophobic motif (*green circle*) are agonist-evoked; this kinase does not have a pseudosubstrate and acute activity is controlled by phosphorylation. *Bottom panel*: for both kinases, phenylalanine (*green*) C-spine mutations are a surrogate for ATP to structure a correctly assembled kinase domain. However, they are catalytically inactive toward substrates and toward hydrophobic motif autophosphorylation. In the case of PKC (*left*), spine mutants have a properly folded kinase domain but are unable to autoinhibit because the hydrophobic motif is not phosphorylated. Without autoinhibition, the activation loop and turn motif sites are phosphatase labile and do not accumulate. This unphosphorylated PKC dimerizes and is dominant negative toward endogenous PKC. In the case of Akt (*right*), spine mutants retain phosphate at the turn motif and activation loop, because hydrophobic motif phosphorylation is not necessary to retain phosphates at these positions; they are not dominant negative. Akt, protein kinase B; PKC, protein kinase C.

In summary, our analysis of four mutations in the kinase domain demonstrates the utility of C-spine mutations in generating catalytically dead kinases while preserving the structurally important hydrophobic core. Our data reveal that a phenylalanine substitution in the C-spine, but not R-spine, is sufficient to inactivate two AGC kinase families: PKC and Akt. The PKC C-spine mutants exhibited loss of phosphorylation at the activation loop, turn motif, and hydrophobic motif, a dominant negative effect over WT PKC, and increased agonist-induced translocation to the plasma membrane, consistent with a more open PKC species (Fig. 7). Despite this open conformation, the two mutants retained protein stability, likely the result of proper assembly of the kinase core. In contrast, the Akt1 C-spine mutants displayed reduced phosphorylation only at the autophosphorylation site, the hydrophobic motif, and were not dominant negative toward endogenous Akt activity (Fig. 7). Despite these subtle differences between two AGC kinase families, our results demonstrate that phenylalanine substitution of C-spine residues blocks ATP binding to generate an inactive kinase while maintaining the closed, active conformation of the kinase domain. These C-spine mutations have also been used to study the scaffolding functions in three TKL family kinases: KSR1, BRAF, and CRAF, showing the applicability of this approach to non-AGC kinases (18, 19). Taken together, these data and the high conservation of the C-spine residues across kinase families support the utility of this novel approach for inactivating protein kinases that can be broadly applied to study the phosphorylation and scaffolding functions of different kinases.

## Experimental procedures

### Plasmids and constructs

The CKAR2, CKAR2 TA, and MyrPalm-CFP were previously described (48). Human, mouse, or rat PKC (βII, γ, θ) and Akt1 were N terminally tagged with HA, mCherry, or YFP in a pcDNA3 vector using Gateway cloning (Life Technologies) as previously described in (49). All mutants were generated using QuikChange site-directed mutagenesis (Agilent) following the manufacturer’s instructions.

### Antibodies and reagents

Vinculin antibody (cat. no. 4650), phospho-PKC (pan) (γ Thr514) antibody (cat. no. 9379), phospho-PKCɑ/βII (Thr638/641) antibody (cat. no. 9375), phospho-PKC (pan) (βII Ser660) antibody (cat. no. 9371), phospho-PKCθ (Thr538) antibody (cat. no. 9377), phospho-PKCδ/θ (Ser645/676) antibody (cat. no. 9376), phospho-Ser PKC substrate antibody (cat. no. 2261), GFP antibody (cat. no. 2956), phospho-Akt (Thr308) antibody (cat. no. 4056), phospho-Akt (Thr450) antibody (cat. no. 121785), phospho-Akt1 (Ser473) antibody (cat. no. 90185), Akt1 antibody (cat. no. 2938), and GAPDH (14C10) antibody (cat. no. 2118) were from Cell Signaling Technologies, Hsp90 antibody (cat. no. 610419) was from BD Biosciences, and HA antibody (cat. no. H9658) was from Millipore and were used at 1:1000 dilution, or at 1:2000 for the phospho-PKCδ/θ (Ser645/676) antibody. ɑ-Tubulin antibody (cat. no. T6074) was obtained from Sigma Aldrich and was used at 1:10,000 dilution. HRP-conjugated anti-rabbit (cat. no. 401315) and anti-mouse (cat. no. 401215) secondary antibodies were from Millipore and used at 1:10,000 dilution. All antibodies were diluted in 1% bovine serum albumin (Millipore, cat. no. 12659) dissolved in PBS-T (1.5 mM sodium phosphate monobasic, 8 mM sodium phosphate dibasic, 150 mM NaCl, and 0.05% Tween-20) with 0.25 mM thimerosal (Thermo Fisher Scientific, cat. no. J61799.14). UTP (cat. no. 6701) and PDBu (cat. no. 524390) were purchased from Calbiochem. Compound 20 (C20) (cat. no. S6577) and GDC0068 (cat. no. S2808) were purchased from Selleckchem. CHX (cat. no. 239763) and CalA (cat. no. 208851) were purchased from Millipore. EGF (cat. no. AF-100-15) was purchased from PeproTech. Bradford reagent (cat. no. 500-0006), protein standards (cat. no. 161-0394), bis/acrylamide solution (cat. no. 161-0156), and polyvinylidene difluoride membrane (cat. no. 162-0177) were purchased from Bio-Rad. Bicinchoninic acid protein assay kit (cat. no. 23225) used for protein quantification was purchased from Thermo Fisher Scientific. Luminol (cat. no. A-8511) and p-coumaric acid (cat. no. C-9008) used to make chemiluminescence solution were purchased from Sigma-Aldrich.

### Cell culture and transfection

COS7 cells were maintained in Dulbecco’s modified Eagle’s medium (DMEM) (Corning, cat. no. 10-013-CV) containing 10% fetal bovine serum (Atlanta Biologicals, cat. no. S11150) and 1% penicillin/streptomycin (Gibco, cat. no. 15-140-122) at 37 °C in 5% CO_2_. Cells were periodically tested for *Mycoplasma* contamination by a PCR-based method (105). Transient transfections were carried out using a Lipofectamine 3000 kit (Thermo Fisher Scientific) per the manufacturer’s instructions, and constructs were allowed to express for 24 h for imaging experiments and CHX assays or for 48 h for phosphorylation assays.

### FRET imaging and analysis

COS7 cells were seeded at 2 × 10^5^ cells per dish into 35 mm dishes (Corning, cat. no. 430165) containing glass cover slips (Fisherbrand, cat. no. 12545102) attached with SYLGARD 184 Silicone Elastomer Kit (Dow, cat. no. 04019862). Cells were transfected 24 h after seeding. For CKAR2 activity assays, cells were cotransfected with mCherry-tagged PKC (see Table 1 for concentrations) and 1 μg CKAR2 or CKAR2 TA DNA (48). For BKAR activity assays, cells were cotransfected with mCherry-tagged Akt1 and 1 μg BKAR DNA for 24 h (57). For translocation assays, cells were cotransfected with 0.75 μg WT or R-spine YFP-PKCβII constructs, 3 μg YFP-PKCβII V356F, or 1 μg YFP-PKCβII A369F and 0.8 μg MyrPalm-CFP. To ensure each dish was transfected with the same amount of DNA, 2.25 μg and 2 μg of YFP vector DNA was supplemented for the wildtype and A369F dishes, respectively. For PKC assays, cells were imaged 24 h post transfection. For Akt assays, cells were serum starved in DMEM for 18 h before imaging. Cells were rinsed once and imaged in 2 ml Hank’s balanced salt solution (HBSS) (Corning, cat. no. 21-022-CV); 1 mM CaCl_2_, diluted from a 1000x from a 1 M CaCl_2_ stock, was added fresh to Hank’s balanced salt solution prior to imaging the PKC experiments. Images were acquired on a Zeiss Axiovert 200M microscope (Carl Zeiss Micro-Imaging Inc) using an Andor iXon Ultra 888 digital camera (Oxford Instruments) controlled by MetaFluor software (Molecular Devices) version 7.10.1.161. Filter sets and parameters for imaging were described previously (106). Background signal was subtracted for each wavelength from an area containing no cells. For PKCθ CKAR2 and PKCβII translocation assays, individual cells were selected for quantification by tracing the entire cell. For PKCβII, γ, and Akt activity assays, individual cells were selected for quantification by tracing the cell, excluding the nucleus. Images were acquired every 15 s, and baseline images were acquired for 3 min prior to drug addition. For PKC activity imaging, agonist/inhibitor stock solutions were prediluted 100x into 200 μl imaging solution, which was then added dropwise to dishes containing 2.0 ml imaging solution, in-between acquisitions, to yield final concentrations of 100 μM UTP, 200 nM PDBu, and/or 1 μM C20. A similar procedure was followed for Akt imaging except agonist/inhibitor solutions were prediluted 50x into 100 μl imaging solution to yield final concentrations of 50 ng/ml EGF and/or 20 μM GDC0068 for Akt imaging. FRET ratios for each cell were normalized to the average of the first or last 3 min as indicated in the figure legends. Data represent mean ± SEM for cells from at least three independent experiments.

**Table 1:** DNA constructs and concentrations

Construct (pcDNA3)	Species	Concentration used (for WB)	Concentration used (for CKAR2/BKAR)
PKCβII WT	Human	0.7 μg	0.75 μg
PKCβII V356F	Human	1 μg	1 μg
PKCβII A369F	Human	1 μg	1 μg
PKCβII L373F	Human	0.7 μg	0.75 μg
PKCβII L394F	Human	0.7 μg	0.75 μg
PKCβII K371R	Rat	1.5 μg	–
PKCγ WT	Human	0.5 μg	1 μg
PKCγ V365F	Human	0.5 μg	1 μg
PKCθ WT	Human	2 μg or 0.8 μg (for CHX assay)	1 μg
PKCθ V394F	Human	2 μg	2 μg
PKCθ A407F	Human	2 μg	2 μg
PKCθ R145H	Human	0.8 μg	–
PKCθ T538A (AL mutant)	Human	2 μg	–
PKCθ S676A (TM mutant)	Human	2 μg	–
PKCθ S695A (HM mutant)	Human	2 μg	–
Akt1 WT	Mouse (WB); Human (BKAR)	1 μg	1 μg
Akt1 V164F	Mouse (WB); Human (BKAR)	1 μg	1 μg
Akt1 A177F	Mouse (WB); Human (BKAR)	0.8 μg	1 μg

AL, activation loop; HM, hydrophobic motif; TM, turn motif.

### Cell lysis and immunoblotting

Cells were washed with Dulbecco’s phosphate-buffered saline (Corning, cat. no. 21-031-CV) and lysed in phosphate lysis buffer pH 7.5 containing 50 mM Na_2_HPO_3_, 1 mM Na_4_P_2_O_7_, 20 mM NaF, 2 mM EDTA, 2 mM EGTA, and 1% Triton X-100 for PKCβ and θ experiments. For PKCγ and Akt experiments, cells were lysed in Tris lysis buffer pH 7.4 containing 50 mM Tris, 10 mM Na_4_P_2_O_7_, 50 mM NaF, 5 mM EDTA, 100 mM NaCl, and 1% Triton X-100. Lysis buffer was supplemented with 50 μg/ml leupeptin, 1 mM PMSF, 1 mM Na_3_VO_4_, 2 mM benzamidine, 1 mM DTT, and 1 μM microcystin prior to lysis. Whole-cell lysates were collected by scraping and briefly sonicated prior to protein quantification by Bradford assay or bicinchoninic acid assay. Samples were boiled in sample buffer containing 250 mM Tris HCl, 8% (w/v) SDS, 40% (v/v) glycerol, 80 μg/ml bromophenol blue, and 2.86 M β-mercaptoethanol for 5 min at 95 °C. Subsequently, 20 to 30 μg protein per sample was analyzed by SDS-PAGE using 7 to 8% acrylamide gels. Gels were transferred to polyvinylidene difluoride membranes by wet transfer method at 4 °C for 2 h at 80 V in transfer buffer (200 mM Glycine, 25 mM Tris–HCl, and 20% methanol). Membranes were blocked in 5% milk for 30 min or 5% bovine serum albumin dissolved in PBS-T for 1 h at room temperature. Membranes were then washed with PBS-T for 5 min three times and incubated with primary antibody overnight at 4 °C with rocking. Membranes were washed for 5 min three times in PBS-T, incubated in the appropriate secondary antibodies for 1 h at room temperature, washed for 5 min three times in PBS-T, and developed with chemiluminescent substrate (100 mM Tris–HCl (pH 8.5), 1.25 mM luminol, 198 μM coumaric acid, and 1% H_2_O_2_) on an Azure 300 Chemiluminescent Imaging System (Azure Biosystems, cat. no. AZI300-01).

### Western blot analysis

COS7 cells were seeded into six-well plates at 2 × 10^5^ cells per well and incubated for 24 h. For PKC, cells were transfected with 1 μg of YFP-empty or indicated YFP-tagged PKC constructs (see Table 1 for concentrations) and allowed to incubate for 48 h before CalA treatment. Cells were treated with 50 μM of CalA or DMSO control for 10 min prior to lysis. For PKCθ, the corresponding phosphorylation site alanine mutant was included as a control for antibody specificity. For Akt, COS7 cells were seeded into six-well plates at 1.3 × 10^5^ cells per well and incubated for 24 h before transfecting with the indicated HA-Akt constructs (see Table 1 for concentrations). Twenty-four hours after transfection, cells were serum starved in DMEM for 15 h and treated with 50 ng/ml of EGF for 8 min prior to lysis. Total and phosphorylated PKC and Akt were analyzed by western blot.

### CHX assay

COS7 cells were seeded into six-well plates at 2 × 10^5^ cells per well. After 24 h, cells were transfected with the indicated HA-tagged PKCθ constructs (see Table 1 for concentrations) and allowed to incubate for 24 h before CHX treatment. Cells were treated with 355 μM CHX, diluted from a stock concentration of 500 mM, or DMSO control, lysed at the indicated times, and analyzed by western blot.

### Kinase modeling

The two C-spine residues, V394 and A407, were mutated to F394 and F407, respectively, using Pymol (Schrödinger, LLC) *in silico*. The crystal structure of Protein Kinase C theta (Protein Data Bank (PDB): 5F9E) was used, and the inhibitor compound was removed from the ATP-binding pocket.

### Quantification and statistical analysis

For imaging experiments, intensity values and FRET ratios were acquired using MetaFluor software and normalized as described above. Western blots were quantified by densitometry using ImageJ version 2.1.0/1.53c. Statistical tests described in figure legends were performed using Prism (GraphPad Software) version 9.5.0. Structures were modeled using PyMOL version 2.3.0 (Schrödinger, LLC).

## Data availability

The findings of this study are supported by the data within the article.

## Supporting information

This article contains supporting information (76).

## Conflict of interest

The authors declare that they have no conflicts of interest with the contents of this article.
