# Supplementary material for: C-spine mutations of protein kinase C and Akt as a novel generalizable approach to create stable pseudokinases
Source: J Biol Chem. 2025 Nov 7;301(12):110921. doi: 10.1016/j.jbc.2025.110921 (PMC12721161; doi:10.1016/j.jbc.2025.110921)
Supplement: Supplementary Figures [file mmc1.pdf]

Supplementary Figure 1

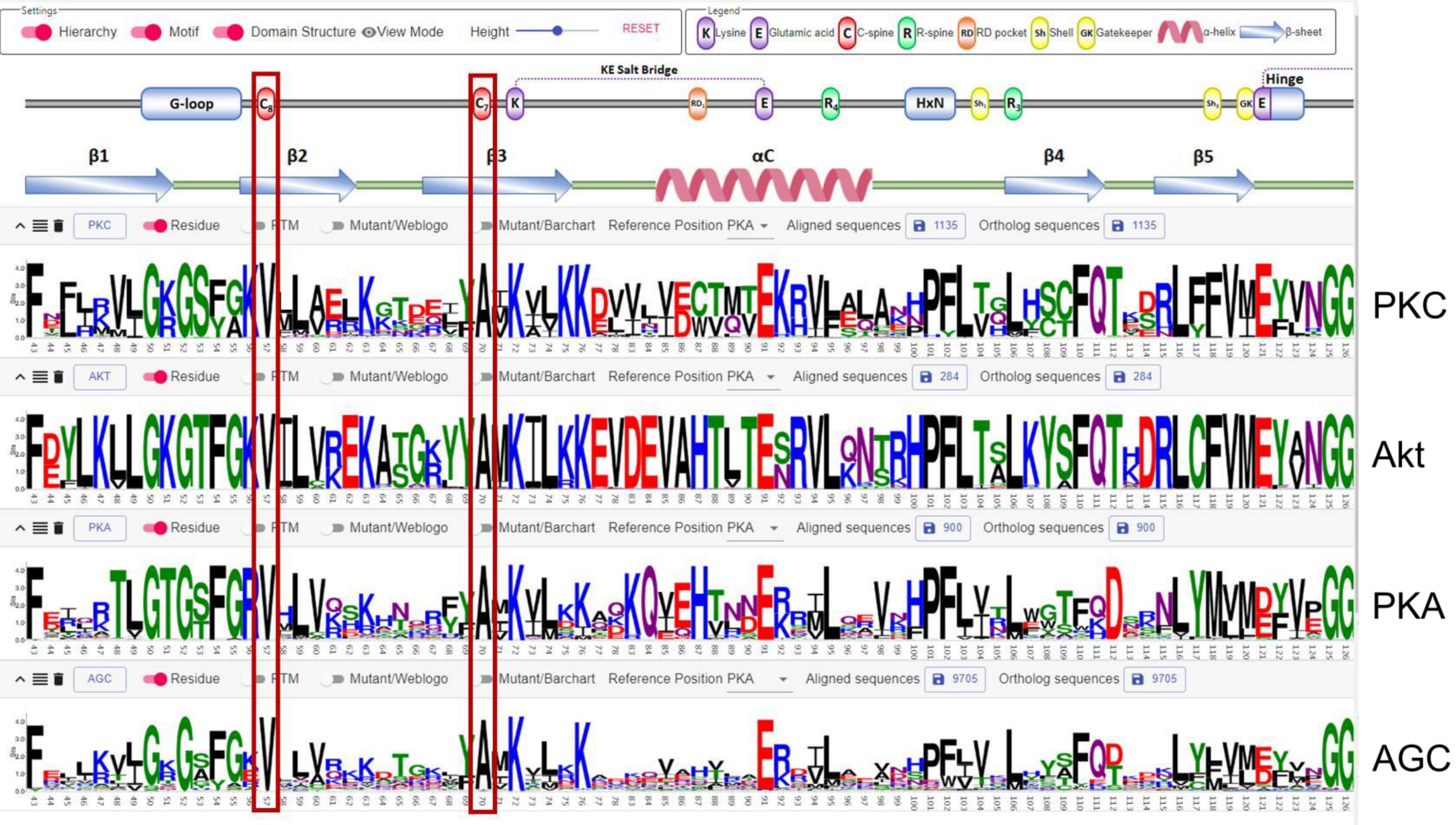

Supplementary Figure 1. C-spine residues are highly conserved across AGC kinases. Alignment of PKC, Akt, and PKA with other AGC family kinases with Kinome Viewer (KinView) (76) illustrates high conservation of C-spine residues V57 and A70 (PKA numbering). This underscores the potential for C-spine mutations to prevent catalysis but maintain scaffold functions in the study of other AGC family kinases.
